# Editorial: Genetics and epigenetics of melanoma and non-melanoma skin cancer

**DOI:** 10.3389/fgene.2026.1770737

**Published:** 2026-01-23

**Authors:** Chiara Moltrasio, Paola Maura Tricarico, Muhammad Suleman, Sergio Crovella, Maurizio Romagnuolo

**Affiliations:** 1 Dermatology Unit, Fondazione IRCCS Ca’ Granda Ospedale Maggiore Policlinico, Milan, Italy; 2 Department of Pediatrics, Institute for Maternal and Child Health - IRCCS “Burlo Garofolo”, Trieste, Italy; 3 Department of Biomedical Sciences, Health Cluster, Qatar University, Doha, Qatar; 4 Department of Environmental and Prevention Science, University of Ferrara, Ferrara, Italy; 5 Department of Medical Biotechnologies, University of Siena, Siena, Italy

**Keywords:** differentially expressed genes, epigenetics, melanoma skin cancer, prognostic model, targeted treatments

The field of genetics and epigenetics of melanoma and non-melanoma skin cancer is rapidly evolving, driven by advancements in next-generation sequencing (NGS) technology. Recent studies have unveiled the complex genomic landscape of cutaneous malignancies, characterized by different pathogenic variants/genetic aberrations, thus providing valuable insights into the biological heterogeneity of these diseases with critical implications for prognosis and therapy. Alongside the genomic landscape, epigenetics - defined as a complex of nongenomic mechanisms that modulate gene expression without changing the genome sequence - has taken on an increasingly important role in the pathogenesis of these skin cancers. Indeed, epigenetic regulators such as DNA methylation, various histone modifications and noncoding RNAs have been identified as crucial players in the initiation and progression of cutaneous malignancies. Nevertheless, research into these individual contributors and how they converge remains fragmented and incomplete, highlighting the urgent need for comprehensive investigations that leverage cutting-edge technologies to elucidate the interplay of genetics and epigenetics underlying these diseases. These in-depth investigations will enable clinicians and researchers to identify predisposing genetic factors, biomarkers for disease progression and patient outcomes as well as novel potential targeted therapies (to be introduced in clinical practice also in combination with existing treatments like immunotherapy).

This Research Topic, mainly comprising original research, discuss novel evidence on the genetics (novel pathogenic variants associated with disease risk and progression) and epigenetics (e.g., regulatory mechanisms driven by noncoding RNAs) of both melanoma and non-melanoma skin cancers. It also reflects the great potential of new technologies such as high-throughput omics and computational frameworks to uncover novel molecular mechanisms and potential therapeutic targets.

To identify druggable targets in cutaneous melanoma (CM), Xing et al. integrated multi-omics Mendelian randomization pipeline, identifying EPS15L1 (Epidermal Growth Factor Receptor Pathway Substrate 15 Like 1) and HGS (Hepatocyte Growth Factor-regulated tyrosine kinase Substrate) as causative plasma proteins increasing CM risk. The expression regulation pattern of genes encoding these proteins was also closely associated with the immune cell infiltration level, playing an important role in the immune microenvironment. Additionally, the identified interactions between EPS15L1 and HGS and doxorubicin - a chemotherapy drug that disrupt DNA function, leading to cell cycle arrest and programmed cell death in rapidly dividing cancer cells -, underscored the potential for drug repurposing of these targets for CM clinical management.

Among the various factors that influence prognosis in melanoma, advanced age is considered an independent poor prognostic factor. Over the last 30 years, there has been an improvement in mortality rate among young adults diagnosed with malignant melanoma (MM), while mortality in older adults has remained stable. Some authors believe that this discrepancy is due to interindividual variability in biological and molecular profiles. To clarify the importance of somatic mutation profiles by NGS in Turkish individuals, Ekinci et al., outlined the distribution of somatic genetic variations in 103 geriatric MM cases compared to that of young adult patients. The observed age-related differences in mutation profiles, affecting the following genes: *NRAS*, *KIT*, *KRAS*, *CDKN2A*, *PTEN*, reinforce the need for age-specific molecular considerations into clinical workup to ameliorate outcome and provide novel personalized therapeutic approaches.

Always considering the high mortality rate of melanoma, Hu et al. began to “construct” a prognostic model associated with glutamine metabolism via bioinformatic approaches, with the goal of developing novel targeted therapies and providing valuable information regarding prognosis management. Similarly, Feng et al. presented a robust prognostic model for CM based on genes associated to PANoptosis, - a newly identified programmed cell death pathway -. From twenty-six differentially expressed genes (DEGs) related to PANoptosis, seven (*CD8A*, *ADAMDEC1*, *CD69*, *CRIP1*, *LSP1*, *BCL11B*, and *CCR7*) were identified as independent prognostic factors. Interestingly, the activity of these prognostic genes was regulated by different microRNAs such as hsa-miR-330-5p and long noncoding RNAs like AL355075.4, reinforcing the strict interplay between genetic and epigenetic factors underlying melanoma skin cancer.

Likewise, Shi et al., developed a risk score model based on DEGs in CM patients undergoing radiotherapy (RT) to evaluate the effects of RT-related genes on several factors including prognosis and response to therapy. The main findings encompassed the crucial role of four RT-related genes (*DUSP1*, *CXCL13*, *SLAMF7*, and *EVI2B*) in predicting prognosis of CM and driving personalized therapies, especially in the context of immunotherapy.

In the field of CM risk factors prediction, machine learning approaches are also rapidly growing, supporting novel scenarios for diagnosis (in terms of early detection), patient outcomes/stratification and personalized therapies based on “private” genetic variants and environmental/epigenetic factors. By combining clinico-epidemiological and computational approaches, Li et al. identified diverse risk factors such as sunburn, older age, Caucasian ethnicity, education level, marital status, smoking, diabetes and hypertension. In addition to these, the authors found that higher vitamin D serum levels decreased melanoma risk, showing a protective effect, mainly in females and younger individuals, thus contributing to the growing body of evidence supporting the chemoprotective role of vitamin D in skin cancer.

Another study conducted by Zhao et al. through a Mendelian randomization study, assumed that higher levels of glycated hemoglobin (HbA1c) may be associated with a decreased risk of melanoma, whereas type II diabetes was associated with a reduced basal cell carcinoma risk. Nevertheless, no evidence has been found to support the association between antidiabetic drugs and skin cancer.

Collectively, the articles in this Research Topic illustrate how the implementation of high-throughput omics and computational analyses is redefining the translational study of melanoma across research and clinical practice, from molecular insights to potential implementation in clinical workup. These contributions underscore the urgent need to integrate in-depth investigations into clinical practice, to ameliorate prognosis and promote novel potential personalized therapeutic strategies.

